# Prebiotic Cellulose–Pullulan Matrix as a “Vehicle” for Probiotic Biofilm Delivery to the Host Large Intestine

**DOI:** 10.3390/polym16010030

**Published:** 2023-12-20

**Authors:** Irina Savitskaya, Sirina Zhantlessova, Aida Kistaubayeva, Ludmila Ignatova, Dina Shokatayeva, Yuriy Sinyavskiy, Almagul Kushugulova, Ilya Digel

**Affiliations:** 1 Faculty of Biology and Biotechnology, Al-Farabi Kazakh National University, Almaty 050040, Kazakhstan; irasava_2006@mail.ru (I.S.); ignat_lv@mail.ru (L.I.); dina_ibrayeva_91@mail.ru (D.S.); 2 Kazakh Academy of Nutrition, Almaty 050008, Kazakhstan; sinyavskiy@list.ru; 3 Laboratory of Human Microbiome and Longevity, Center for Life Sciences, National Laboratory Astana, Nazarbayev University, 53 Kabanbay Batyr Avenue, Astana 010000, Kazakhstan; akushugulova@nu.edu.kz; 4 Institute for Bioengineering, Aachen University of Applied Sciences, Heinrich-Mußmann-Straße 1, D-52428 Jülich, Germany; digel@fh-aachen.de

**Keywords:** *Lactobacillus rhamnosus* GG, biofilms, bacterial cellulose, prebiotic, immobilization

## Abstract

This study describes the development of a new combined polysaccharide-matrix-based technology for the immobilization of *Lactobacillus rhamnosus* GG (LGG) bacteria in biofilm form. The new composition allows for delivering the bacteria to the digestive tract in a manner that improves their robustness compared with planktonic cells and released biofilm cells. Granules consisting of a polysaccharide matrix with probiotic biofilms (PMPB) with high cell density (>9 log CFU/g) were obtained by immobilization in the optimized nutrient medium. Successful probiotic loading was confirmed by fluorescence microscopy and scanning electron microscopy. The developed prebiotic polysaccharide matrix significantly enhanced LGG viability under acidic (pH 2.0) and bile salt (0.3%) stress conditions. Enzymatic extract of feces, mimicking colon fluid in terms of cellulase activity, was used to evaluate the intestinal release of probiotics. PMPB granules showed the ability to gradually release a large number of viable LGG cells in the model colon fluid. In vivo, the oral administration of PMPB granules in rats resulted in the successful release of probiotics in the colon environment. The biofilm-forming incubation method of immobilization on a complex polysaccharide matrix tested in this study has shown high efficacy and promising potential for the development of innovative biotechnologies.

## 1. Introduction

Many therapeutic or preventive actions implemented to address disorders of the intestinal microbiome base on or benefit from the use of probiotics. The idea is that the administration of exogenous probiotics may help to restore and strengthen the local intestinal microbiota, which, as an integral part of the mucosal barrier, largely contributes to maintaining the health of the whole gastrointestinal (GI) tract and to its resistance to pathogen colonization [1,2]. For this purpose, the introduced probiotics must successfully, i.e., without significant losses, reach the large intestine and (at least transiently) colonize its mucosa to work in it. 

Immobilization is a well-known method to provide protection to sensitive substances and cells in harsh environments, rendering them more suitable for a range of sectors, such as tissue engineering, cosmetics, drug delivery, and environmental applications [3]. Thus, the system carrier + immobilized probiotic is a reasonable design for the goal of safe delivery of probiotics to the target organ (the large intestine) after having gone through aggressive environments of the stomach and the small intestine. 

A simple and cost-effective technological method of probiotic immobilization is the inclusion of biopolymers in the gel’s spatial structure, known as membrane capture or the adhesive method [4]. This method is based on the natural phenomenon since many microorganisms in their natural habitat form biofilms through initial attachment at liquid–solid interfaces [5]. Biofilms are defined as matrix-enclosed microbial populations that adhere to each other and/or to surfaces [6]. Biofilm endows cells with many beneficial properties, including reduced cell leaching, enhanced biochemical stability, promoted cell-to-cell interaction through quorum sensing, and improved substrate distribution [7,8]. Epithelial associations formed by resident representatives of the intestinal tract also exhibit characteristics typical of bacterial biofilm formation [9]. The bacteria included in the biofilm are firmly attached to the surface of nonglandular squamous stratified epithelium and simultaneously are embedded in a self-producing matrix of extracellular polymeric substance [10,11]. Life within an intestinal biofilm is a selective advantage that allows microorganisms to exist in a protected niche and directly interact with the host.

Since not all probiotic microorganisms can form biofilms, their artificial enforced encapsulation into provided biofilms could facilitate and promote the colonization and retention of beneficial strains in the GI tract, thereby facilitating their probiotic properties [12].

Polysaccharide gels are presumably the most suitable biofilm matrix material for probiotic immobilization, as such hydrogels are very resistant to stress conditions during processing and transit through the GI tract [13,14].

In our study, the use of bacterial cellulose (BC) seems promising as the carrier base of the matrix. Along with its high porosity and strength, BC has a high adsorption capacity and is fully biocompatible with both microorganisms and the human body [15]. The immobilization of microbial cells on BC, including probiotics, has been quite successfully carried out for a variety of purposes [16,17,18].

While biofilm immobilization provides a physical protective barrier against external stresses [19], an additional option can be used to enhance cell resilience and viability. This involves including into the matrix an additional polysaccharide component, having prebiotic activity. Prebiotics are indigestible polysaccharides, which selectively nourish saccharolytic probiotic microorganisms [20]. As a result, the presence of the prebiotic in the matrix will stimulate the proliferation of probiotic cells, which will promote the formation of a probiotic biofilm on it, providing additional protection.

The formation of biofilms is affected by various factors, with one of the determining factors being the composition of the nutrient medium in which the bacterial culture is grown [21]. One of the objectives of this study is to determine the effect of the nutrient medium components inducing the biofilm phenotype.

*Lactobacillus rhamnosus* GG (LGG), a probiotic production strain known for its beneficial effects on human health [22], will be immobilized into the combined polysaccharide matrix. This strain can form biofilm-like communities on abiotic surfaces [23]. This research examines the opportunity to immobilize not planktonic probiotic cells but biologically active probiotic biofilms.

The matrix will be obtained through microbial biosynthesis by cocultivating two producers: *Komagataeibacter xylinus*—BC and *Aureobasidium pullulans*—pullulan (PUL) [24]. PUL serves as a prebiotic for lactobacilli [25] and LGG [24]. It is assumed that this synbiotic system (neutral carrier + prebiotic + probiotic) can be used to deliver the “working strain” to the target niche—the large intestine. This study reports for the first time high-density biofilm cells immobilized in BC/PUL granules by the membrane-capture method in a biofilm-inducing medium.

The purpose of the work is to study the possibility of using a polysaccharide matrix with probiotic biofilms (PMPB) as a transport system for probiotics introduced into the host organism.

## 2. Materials and Methods

### 2.1. Preparation of the BC/PUL and BC Films

BC/PUL material was obtained by cocultivating BC and PUL producers—*K. xylinus* C3 and *A. pullulans* C7, respectively. *K. xylinus* C3 and *A. pullulans* C7 strains were isolated at the Biotechnology Department, Al-Farabi Kazakh National University, and deposited in the Republic Collection of Microorganisms (Astana, Kazakhstan). 

To obtain the inoculum of *K. xylinus* C3, several colonies were transferred from an agar plate to 100 mL of the Hestrin–Schramm (HS) broth medium in order to obtain a cell concentration of about 10^5^ CFU/mL. The inoculum of *A. pullulans* C7 was obtained in a similar manner using 25 mL of a Chapek Dox medium (HiMedia, Mumbai, India). Next, both of the obtained 24 h inocula (1%, *v*/*v*) were added to a flask containing 100 mL of a molasses medium. The composition of the molasses medium (g/L) was molasses—20, Na_2_HPO_4_—2.7, KH_2_PO_4_—1, peptone—5, yeast extract—5, citric acid—1.15, ethanol—10, MgSO_4_·7H_2_O—0.5, KCl—0.5, and FeSO_4_—0.01. All reagents and media supplements were purchased from Veld Co. (Almaty, Kazakhstan). Cultivation in the molasses medium was carried out statically in 500 mL Erlenmeyer flasks at 30 °C for 7 days. 

To obtain the BC film, the inoculum of *K. xylinus* (1%, *v*/*v*) was added to a flask containing 100 mL of an HS broth medium. The incubation conditions were the same as those described above.

The resulting films were purified by washing with deionized water, treated with 1% (*w*/*v*) NaOH at 35 °C for 24 h to eliminate microbial cells, and rinsed again with deionized water. Finally, the samples were dried in an oven at 60 °C to a constant weight. Each preparation step was performed in triplicate. The morphostructural features of the obtained films were described in our previous study, where the inclusion of PUL in BC was proved by scanning electron microscopy (SEM) and by Fourier transformed infrared spectroscopy [24]. 

### 2.2. Probiotic Bacteria and Growth Conditions

*Lactobacillus rhamnosus* GG (ATCC^®^ 53103^TM^) was purchased from the American Type Culture Collection. The strain was cultured in MRS medium (HiMedia, Mumbai, India) at 37 °C for 48 h to obtain a cell concentration of 10^8^ CFU/mL. Subsequently, the cells were harvested using a laboratory centrifuge, RS-6MC (Dastan, Bishkek, Kyrgyzstan), at 4000× *g* for 15 min and washed twice with isotonic saline. 

The compositions of the media were, for the MRS broth (g/L), peptone—10, meat extract—8, yeast extract—4; glucose—20, K_2_HPO_4_—2, Tween-80—1, disubstituted ammonium citrate—2, sodium acetate—5, MgSO_4_—0.1, and MnSO_4_—0.05 (pH 6.5); and for the PYG broth (g/L), bacteriological peptone—15, casein enzymatic hydrolyzate—5, glucose—10, and yeast extract—10 (pH 6.5).

Small modifications of the concentrations of some components or the introduction of additional ones are specified later in the results paragraph. 

### 2.3. LGG Biofilm Formation and Its Analysis by the Microplate Method

The method proposed by Lebeer et al. [23] was used with minor modifications, as follows: 200 μL of a nutrient medium and LGG suspension (10^8^ CFU/mL in saline) were mixed in a polystyrene microtiter plate well and incubated for 48 h at 37 °C. 

The biofilms formed in wells were washed 5 times with 200 µL of phosphate-buffered saline (PBS) and dried at 25 °C for 40 min in an inverted position. The remaining attached bacteria were stained with 1 mL of 0.1% (*w*/*v*) crystal violet (Merck, Darmstadt, Germany) for 30 min. After staining, the dye solution was aspirated, and the wells were washed 5 times with 200 µL of distilled water and dried for 30 min at 25 °C. The dye associated with the adherent biofilm was extracted with 200 μL of a mixture of ethanol and acetone (80:20). Then, 150 μL aliquots were taken from each well, and the optical density (OD) was determined at 570 nm. A sterile culture medium was used as a negative control. Biofilm formation results were classified as positive (OD 570 ≥ 1), weak positive (0.1 ≤ OD 570 < 1), or negative (OD 570 < 0.1).

To study the possibility of an LGG strain forming a biofilm on the carrier materials, polysaccharide disks were placed in the wells of the plate, and each well was filled with a nutrient medium inoculated with bacteria (10^8^ CFU/mL). The plate was then incubated for up to 48 h at 37 °C, with parafilm sealing to prevent evaporation. Biofilm formation efficiency was analyzed as described above. Additionally, the presence or absence of the biofilm was confirmed by SEM.

### 2.4. Preparation of Granules with Probiotic Biofilms

A suspension of LGG bacteria with a cell concentration of 10^8^ CFU/mL was used for the immobilization in BC and BC/PUL granules. Granules were obtained by grinding BC and BC/PUL films in an IKA MultiDrive basic mill (IKA, Staufen, Germany). Sterilized granules and bacteria cells were placed in PYG_m_ medium designed to stimulate biofilm formation, and incubated at 37 °C for up to 48 h to ensure the proper immobilization into the carriers. Then, the granules were rinsed from weakly attached cells with sterile saline.

The number of immobilized bacteria was determined by enumerating bacterial cell suspension obtained after digestion with cellulase on MRS agar using the pour plate count method (as described below).

### 2.5. Microscopy

The microscopic examinations used in this study included SEM, laser confocal fluorescence microscopy, and conventional light microscopy.

The microstructure of the samples was observed using SEM modules JSM-7800F (Jeol, Tokyo, Japan) and Zeiss Supra 55VP (Zeiss, Jena, Germany). Dried samples were mounted on a metal stub, sputter-coated with platinum–palladium alloy (Pt/Pd 80/20), and analyzed under different magnifications at an accelerating voltage of 5 kV.

For fluorescence microscopy, a working solution of dye was prepared by adding 3 μL of SYTO^®^ 9 stain, LIVE/DEAD™ BacLight™ Bacterial Viability Kit (Invitrogen, Thermo Fisher Scientific, Waltham, MA, USA) to 1 mL of PBS. An amount of 200 μL of staining solution was added to a sample, incubated for 15 min at room temperature protected from light, and finally rinsed with PBS. Samples were observed with a Biozero fluorescence microscope (Keyence, Osaka, Japan, excitation—480 nm; emission—500 nm). 

### 2.6. Examination of Tolerance to Gastric Acid and Bile Salts

An amount of 5 g of the obtained PMPB granules were subjected for 2 h to 50 mL of artificial gastric juice (AGJ) consisting of 3 g/L pepsin dissolved in sodium chloride solution (2 g/L) adjusted to pH 2 with 0.1 mol/L hydrochloric acid to simulate the stomach conditions. After this step, the same granules were also placed into an artificial bile juice (ABJ) (0.3% solution of bile salts) for 2 h. Both solutions were previously sterilized by filtering through a 0.22 μm membrane. All reagents were purchased from Veld (Almaty, Kazakhstan). The tests were performed at 37 °C to mimic the body temperature.

Survived bacteria were enumerated by pour plate counts on MRS agar after incubation at 37 °C for 72 h. The survival degree of LGG was expressed as log CFU/g. 

To evaluate the survival of free cells, 1 mL of bacterial suspension was introduced into 9 mL of AGJ and incubated (for 2 h). Subsequently, 1 mL of the suspension was collected and transferred into 9 mL of ABJ solution. The incubation conditions for the free bacteria were identical to those used for the granules. After the incubation period, the survival of the free cells was determined using the aforementioned method.

### 2.7. Release of Probiotic into Artificial Colon Juice (ACJ) with Fecal Suspension/Enzymatic Fecal Extract

Fresh fecal samples were provided by three donors (two females and one male, aged 22–30). All donors neither had GI diseases nor took pro- or prebiotics or antibiotics within 3 months before the sample collection. 

The fecal sample from each donor was diluted with sterile potassium phosphate buffer (0.01 M, pH 7.4) and blended using a Mini-Beadbeater (BioSpec, Bartlesville, OK, USA) to receive 10% (*w*/*v*) fecal slurry. Then, it was centrifuged (2000× *g*, for 5 min), and the supernatant was used as a fecal suspension. To prepare the fecal enzymatic extract, the supernatant after the first centrifugation was additionally centrifuged at 10,000× *g* for 20 min. The supernatant (fecalase) extracted after the second centrifugation was additionally filtered through a 0.22 μm membrane to remove fecal microflora. 

After 2 h incubation in ABJ as described above, PMPB granules (5 g) were placed in 45 mL of freshly prepared ACJ (5 mL of fecal suspension or fecal enzymatic extract mixed with 0.2 g/L of potassium chloride, 8 g/L of sodium chloride, 0.24 g/L of potassium phosphate monobasic, 1.44 g/L of sodium phosphate dibasic, pH 7.2). All experimental groups were incubated for 3–18 h in a shaker incubator at anaerobic (for fecal suspension) or aerobic (for fecal enzymatic extract) conditions at 37 °C. At the end of the incubation period, 1 mL aliquots were collected, and released bacteria were enumerated by pour plate counts in MRS agar, after incubation at 37 °C for 72 h.

### 2.8. Cellulase Activity Assays

#### 2.8.1. Agar Plate-Based Method

The method described by Balla A. et al. [26] was modified and used to compare the cellulolytic activities of the fecal enzyme extract and fecal suspension. The assay was carried out by adding 100 µL of samples to the wells containing carboxymethyl cellulose (CMC) (1%, *w*/*v*) agar medium and incubating at 30 °C for 24 h. Congo red solution (1%, *v*/*v*) was used to reveal the zone of cellulose hydrolysis that forms around the well after the addition of the solution (15 min), followed by discoloration with a NaCl solution (1 M) for 10 min. The diameter of the clear zones surrounding the wells was measured to evaluate the cellulase activity. The enzymatic index E_i_ was determined using the following equation:

E_i_ = (diameter of zone − well diameter)/well diameter

#### 2.8.2. Dinitrosalicylic Acid (DNS) Method

The cellulase activity in the fecal suspension/enzyme extract was assessed using the DNS (3,5-dinitrosalicylic acid) method [27], which measures the quantity of reducing sugars released during hydrolysis. A 1% solution of CMC was prepared in 1 M citrate buffer with a pH of 5.0, and it served as the substrate. To initiate the reaction, 100 µL of the probe and 1 mL of citrate buffer were added to 1 mL of the CMC solution. The mixture was then incubated at 45 °C for 30 min. Next, 1 mL of DNS was introduced to halt the reaction. The treated samples were boiled for 10 min and cooled in a water bath for color stabilization, and the OD was measured at 540 nm. One activity unit was defined as the enzyme amount capable of hydrolyzing CMC and releasing 1 µmol of reducing sugars per min.

### 2.9. In Vivo Study in Rats

#### 2.9.1. Experimental Animals

Outbred male rats weighing 180–200 g underwent a 1-week acclimation period in the experimental animal facility. They were housed at a controlled temperature of 22 ± 2 °C and 50 ± 15% humidity. 

At 5 weeks of age, the animals were randomly divided into three groups (10 rats each). The first and the second groups were given a powdered diet consisting of a 3:1 mixture of normal commercial diet and PMPB or free LGG suspension (≈9 log CFU per day), respectively, over a period of 7 days. Meanwhile, the control group received only a powdered normal commercial diet. Fecal samples were collected at several time points during a week of treatment and a week of observation. 

#### 2.9.2. Detection of LGG in Rat Fecal Samples

Rat fecal samples were homogenized and diluted 10-fold using a peptone physiological salt solution (Sigma-Aldrich, Taufkirchen, Germany). The fecal suspension was used for bacterial enumeration by plating onto MRS agar and incubating for 72 h at 37 °C under anaerobic conditions. The bacterial counts in feces were expressed as the log CFU/g of feces. For LGG identification, randomly selected colonies from each agar plate were inoculated onto MRS agar containing an indicator dye (0.04 g/L of bromocresol purple), where lactose was the only carbon source. During incubation, lactose-positive bacteria produce lactic acid by lactose fermentation, which reduces the media pH, causing a color change from purple to yellow. In contrast, lactose-negative bacteria form small colonies and utilize peptone and yeast extract for growth without producing lactic acid, resulting in the colonies retaining their purple color. Additionally, bacteria were examined using a light microscope and SEM to confirm that cells had cell shapes typical of LGG (rods in chains). 

All these potential LGG isolates were further identified by the fluorescence in situ hybridization (FISH) technique using a target-specific probe for a 16S rRNA sequence. The fluorescence-labeled probe Lcas467-Cy3 (5′-CCGTCACGCCGACAACAG-3′), which binds LGG, was used for the identification of this strain [28]. The bacterial suspension (≈10^7^ cells) on the glass slides was hybridized by the addition of 10–20 μL of hybridization buffer (0.9 M NaCl, 20 mM Tris HCl (pH 7.2), 0.1% sodium dodecyl sulfate, 30% formamide) with 1 μL of oligonucleotide probe and incubated at 46 °C for 2 h. After hybridization, each sample was washed with a buffer (180 mM NaCl, 20 mM Tris HCl (pH 7.2), 0.1% sodium dodecyl sulfate, 5 mM EDTA). The microscope slides were rinsed with distilled water and air-dried. Then, the samples were imaged using Biozero fluorescence microscopy (Keyence, Osaka, Japan). 

### 2.10. Statistical Analysis

Unless otherwise stated, all the experimental groups were assayed in triplicate. The experimental measurements were presented as the mean and standard deviation (mean ± SD). The difference between the groups was analyzed using one-way analysis of variance (ANOVA), followed by Tukey’s test. All statistical analyses were performed using the SPSS software (version 28.0, IBM Corp., Armonk, NY, USA). A *p*-value of less than 0.05 was considered statistically significant. 

### 2.11. Ethical Information

The study was conducted in accordance with the Declaration of Helsinki, approved by the local ethical committee of the Academy of Preventive Medicine and Kazakh Academy of Nutrition (No. 4; 13 June 2022); followed national and international regulations on animal experimental use and approved by the local ethical committee of Al-Farabi Kazakh National University (No. 36; 19 June 2022). Informed consent was obtained from all subjects involved in the study. 

## 3. Results

### 3.1. Effect of Culture Medium Components on Biofilm Formation by LGG

Many adhesive bacteria, including LGG [29], occur naturally in the form of surface-attached biofilms, where they are enclosed in a self-produced extracellular matrix that protects them from adverse environmental conditions [30]. It seemed appropriate to study the biofilm-forming ability of the LGG strain when it was cultivated on different media since it is known that their composition can affect the ability of probiotic bacteria to form biofilms [23].

**Figure 1:**
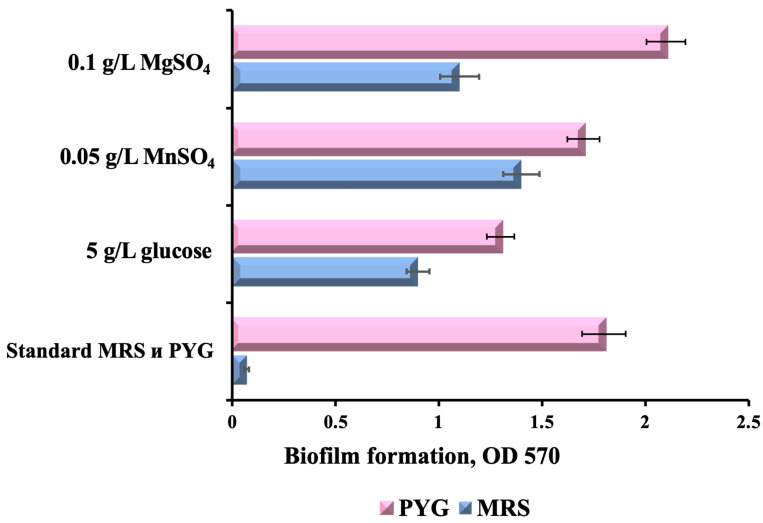
Effect of MRS and PYG media components on the LGG biofilm formation degree. The data are presented as the mean OD with error bars representing the standard deviation.

In this work, for the detection of the biofilm formation, we used a method based on the binding of crystal violet dye to both microbial cells and a biofilm matrix. This method makes it possible to obtain cell density values of a biofilm attached to a microplate well (Figure 1).

On the MRS medium, no LGG biofilm formation was observed with the absorbance level below 0.1, which is identical to the results of the negative control. In contrast, in the PYG medium, which has salt deficiency and lower glucose content (1% instead of 2% in MRS), substantial biofilm formation (OD 570 ≥ 1) was observed after 48 h of incubation. 

The specific influence of glucose on the biofilm formation can be assessed by varying glucose concentrations in the same medium. Glucose, a primary raw material for synthesizing exopolysaccharides (EPS) (the main component of the biofilm matrix) [31], was reduced to 0.5% in one of our experimental variants, resulting in improved biofilm formation compared with standard MRS. However, if the glucose concentration was reduced to 0.5% in the PYG medium, there was a decrease in biofilm formation by LGG. Similarly, additional supplementation of the PYG medium with glucose (up to 2%) also resulted in significantly reduced biofilm formation. This phenomenon clearly demonstrates the relevance of glucose concentration for biofilm formation in both MRS and PYG media. Previously, several other studies also demonstrated that limited nutrient availability promoted biofilm formation, presumably by creating stressful conditions that stimulated the expression of survival-related genes, including those associated with biofilm formation [23,32,33]. The described effect seems to depend on the strain. For instance, Leber et al. [23] demonstrated that the removal of glucose from MRS medium significantly increased the biofilm-forming ability of LGG, while another strain, *L. casei* Shirota, showed almost no reaction. Our data imply that the optimal glucose concentration for biofilm formation by LGG in PYG medium is close to 1%.

It is well known that bacterial adhesion, as the initial and most crucial stage of biofilm formation, is strongly influenced by divalent cations as they determine electrostatic interactions between the contacting surfaces [29]. In our study, the supplementation of glucose-free MRS with MnSO_4_ (0.05 g/L) showed a distinct stimulating effect on biofilm formation, while the addition of MgSO_4_ (0.1 g/L) resulted in a relatively slight increase. The observed difference is likely due to different growth-stimulating activities of these salts. As previously described by Imbert et al., for lactobacilli, including LGG, Mn^2+^ strongly stimulated growth in suspension [34], while no such stimulation was observed for Mg^2+^. As reported by Dertli, the removal of a solution of salts containing Mn^2+^ and Mg^2+^ from MRS medium significantly reduced the formation of *L. johnsonii* FI9785 biofilm and its mutants, possibly due to the essential role of these metal ions in cellular metabolism [35]. We also observed that the introduction of Mn^2+^ into PYG medium did not significantly affect the efficiency of biofilm formation, while the addition of Mg^2+^ stimulated its formation. Thus, among the tested culture media modifications, the optimal for biofilm formation seems to be PYG medium supplemented with 0.1 g/L of MgSO_4_.

Biofilm formation involves EPS synthesis. It has been shown that the level of EPS production, as well as the structural composition, can play a role in the formation of a biofilm [36]. It is usually assumed that complex polysaccharides present in the nutrient medium can either be incorporated into the extracellular matrix or contribute to the proliferation of bacteria, thereby indirectly enhancing the formation of a biofilm. In this regard, for better biofilm formation by LGG, it might be beneficial to supplement the nutrient medium with a polysaccharide that specifically stimulates the growth of this strain. Leber et al. demonstrated inulin’s positive effect on LGG, attributing it to the stimulation of cell aggregation through increased production of EPS [23]. In our experiments, introducing different concentrations (from 0.5 to 5 g/L) of inulin into PYG medium, it was determined that 1 g/L was sufficient and optimal (Table 1). 

A number of researchers who studied the effect of various additives on the effectiveness of biofilm formation by lactobacilli have found that bile, presented in the digestive tract, plays an important role [37,38]. Bile induces the expression of genes encoding the biosynthesis of adhesives and EPS, although this feature correlates with bile resistance in the tested strains [37]. Consequently, we introduced different concentrations of bile in addition to inulin into PYG medium for LGG cultivation. The bile addition increased biofilm development by 1.5–1.9 times.

**Figure 2:**
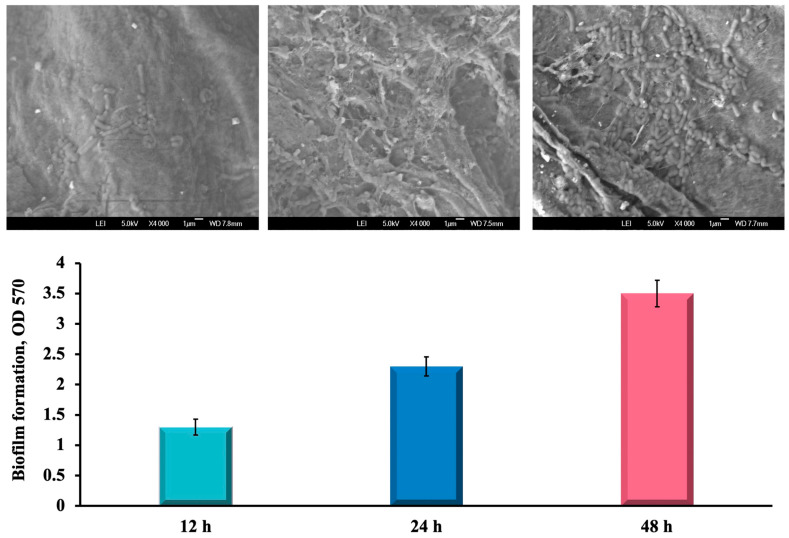
Dynamics of LGG biofilm formation on the BC/PUL polysaccharide matrix with SEM images. The data are presented as the mean OD with error bars representing the standard deviation.

Moreover, these variations of media were added to the wells of the microplate containing BC/PUL disks; i.e., biofilm formation was assessed on the polysaccharide matrix. As a result, it was found that, first, the strain GG was able to form a biofilm on the BC/PUL composite, and second, the addition of 1 g/L of inulin and 0.5 g/L of bile to the medium positively contributed to the formation of biofilms as visualized by electron microscopy (Figure 2). 

Based on the obtained results, the composition of PYG_m_ medium was optimized for biofilm formation by ATCC^®^ 53103^TM^ as follows (g/L): peptone—15, casein hydrolysate—5, glucose—5, yeast extract—10, MgSO_4_—0.1, inulin—1, and bile—0.5.

### 3.2. Immobilization of LGG Cells in Polysaccharide Matrix Granules

The three-dimensional porous network structure of BC, with a large surface area, is composed of nanofibers, capable of holding a large number of inorganic and organic molecules. The presence of hydroxyl groups on the surface of cellulose enables the adsorption of molecules through hydrogen bonding and electrostatic interactions [39]. Therefore, BC may serve as an excellent substrate for cell immobilization and a good matrix support material. 

One of the most effective methods for microorganism immobilization is the “adsorption–incubation” method, as proposed by Nguyen [40]. The main principle is that, during the adsorption stage, bacteria are kept in a saline solution with the carrier, allowing microbial cells to adhere to the surface without an increase in their initial quantity due to the absence of nutrients in the medium. To create conditions for cell reproduction and increase their concentration, the adsorption step is followed by incubation of the carrier with immobilized cells in a nutrient-rich medium. This incubation stage facilitates the reproduction of the already-attached cells both on the surface and inside the carrier, leading to an overall increase in their numbers.

**Figure 3:**
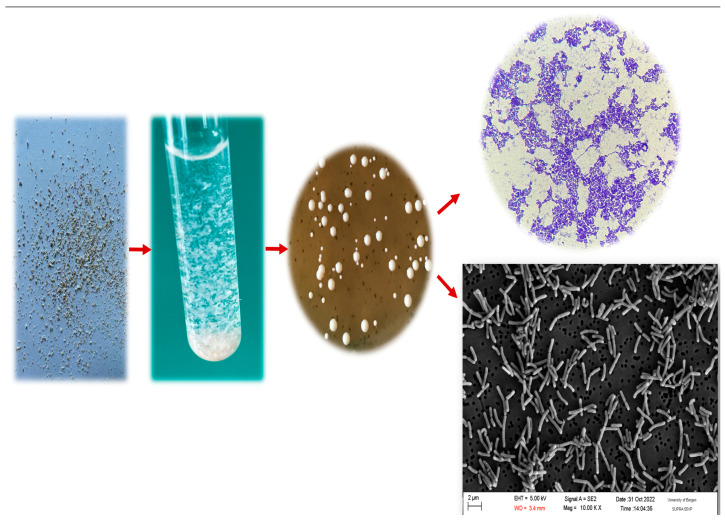
Morphology of isolated colonies and microscopic images of LGG cells after immobilization into BC/PUL granules.

In this study, one of the objectives was to immobilize the probiotic culture as a biofilm. To do this, the LGG inoculum was cultured in PYG_m_ medium, which induces biofilm formation until a concentration of 10^8^ cells/mL was reached. BC and BC/PUL granules, with an average size of 1–2 mm, were introduced into PYG_m_ medium and incubated at a optimal temperature of 37 °C for 48 h to promote biofilm formation. Thus, bacteria were immobilized in polysaccharide matrixes without the explicit need for an adsorption stage. The number of immobilized bacteria was determined by quantitatively plating the culture suspension on MRS agar after treating the granules with the cellulase enzyme (Figure 3). The morphological properties of LGG bacteria remained stable during the whole immobilization process. Microscopically, LGG cells are immobile, rod-shaped, and Gram-positive bacteria. LGG colonies on MRS agar appear white, rounded, and convex, with a smooth and shiny surface and a soft consistency. 

**Figure 4:**
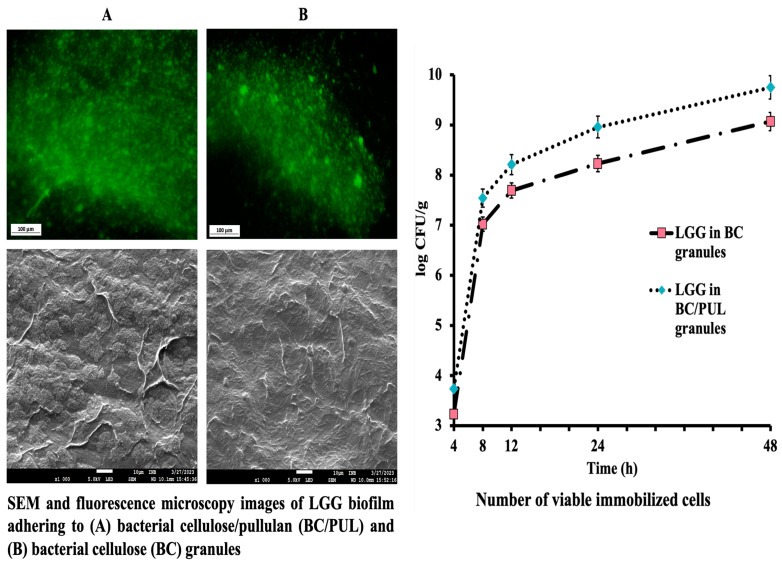
Immobilization efficiency of biofilm-forming LGG in BC and BC/PUL granules.

Incubating granules in PYG_m_ broth for 48 h, to allow biofilm formation, increased cell counts in approximately 1–1.7 log. The average of three assays showed that bacterial counts in BC granules rose from 1.03 × 10^8^ (8.01 log CFU/g) to 1.20 × 10^9^ CFU/g (9.07 log CFU/g), while in BC/PUL to 5.64 × 10^9^ CFU/g (9.75 log CFU/g) (Figure 4). The high volume of bacteria included in both variants of granules indicates that the immobilization process was adequate, and the carrier materials were compatible with the probiotic strain. The structure of cellulose granules is quite “spongy”, so nutritional substances from the environment could diffuse into the internal space of the granules, ensuring the growth and reproduction of strain cells not only on the surface but also inside.

The bacterial cell number was higher when culturing granules with the presence of PUL, where prebiotic apparently allowed bacteria to proliferate faster. All granules, with or without prebiotic, showed the presence of probiotic microcolonies by SEM and fluorescence microscopy (Figure 4). 

As shown on the SEM images, LGG cells dispersed in a cellulose porous network and were embedded in the fibrous matrix. The structure of cellulose, consisting of small pores distributed on the surface, and large pores distributed in the interior can provide an appropriate living space for microorganisms. LGG cells clearly showed dense colonization and a highly compact biofilm structure. The dense biofilm was formed after 48 h of incubation in PYG_m_ broth. These observations were confirmed by viable cell counts. 

The fluorescence microscopy results also demonstrated an intensity in both variants. A strong fluorescence of these bacteria confirms that they are alive probiotic cells. It was observed that the strain GG was able to form a dense biofilm on the biopolymers. The obtained results confirm the successful immobilization of LGG bacteria on the carriers. 

Due to the porous fibrous base of BC, it can be used as a convenient matrix for probiotic biofilms. The immobilization procedure was very simple, inexpensive, and easily realizing and did not require any chemical modifications.

### 3.3. Survival of Free and Immobilized Bacteria under In Vitro Acidic and Bile Salt Conditions

An important parameter to assess the protective effect of the developed probiotic-based vehicles is their ability to tolerate the harsh conditions of the upper GI tract. 

Although the ultimate model for determining the functional effectiveness of such a “vehicle” is the human body, this approach has ethical limitations. Therefore, most studies use the “artificial GI tract” model that simulates the physicochemical state of the main parts of the digestive system—the stomach and the small and the large intestines [41,42]. Such in vitro model is specifically designed to simulate the passage of the immobilized LGG through the different parts of the GI tract. 

**Figure 5:**
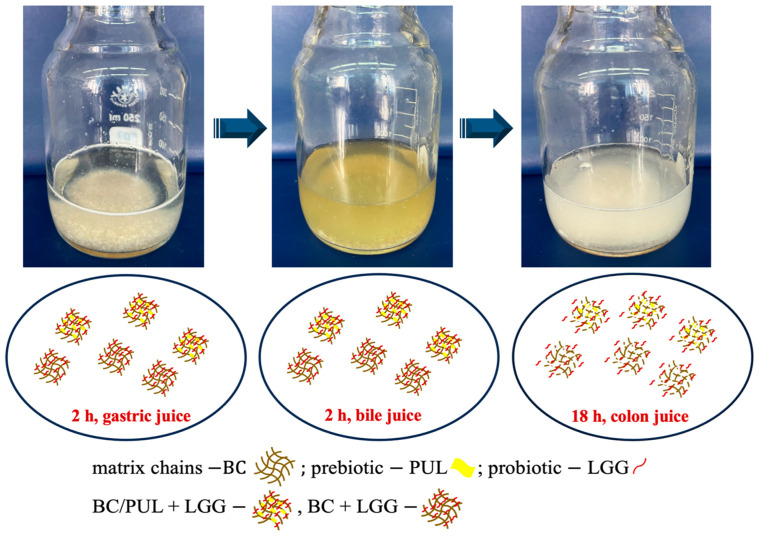
Experimental design of the artificial GI tract.

In this part of the study, we assessed and compared the protective effect of LGG immobilization in BC and BC/PUL granules in the presence of GI fluids (Figure 5). 

As the probiotics reach the stomach, they are exposed to the highly acidic gastric fluid, which poses a highly harsh environment for most bacteria. Furthermore, the stomach poses additional conditions unfavorable for probiotics, such as high ionic strength, protease activity (pepsin), and mechanical churning [43].

**Figure 6:**
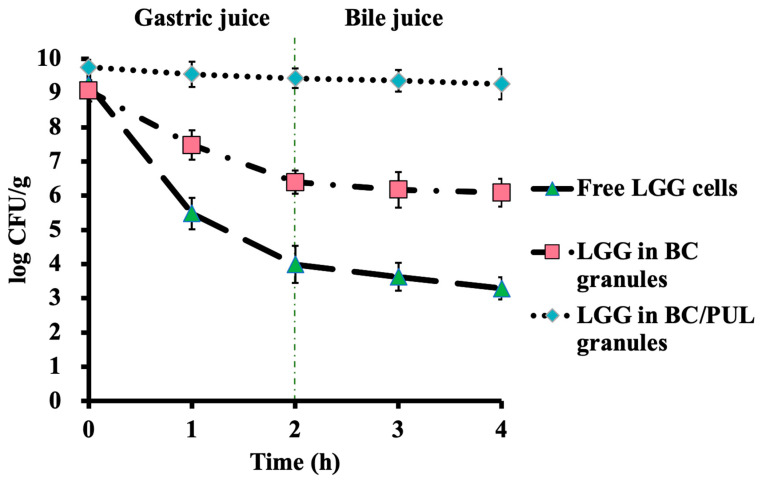
Survival of free and immobilized LGG cells in the sequential incubation during artificial gastric and bile juice treatment.

LGG cells in a BC/PUL composite survived very well after exposure to acidic conditions (pH 2.0) for 2 h compared with the other groups (BC granules, free cells). Figure 6 shows the results for the viability of LGG exposed to AGJ conditions for the free cells, immobilized ones, and also those coimmobilized along with PUL. In BC granules, 6.39 log CFU/g survived the 2 h AGJ exposure. According to the findings reported by Jagannath et al. [44], BC forms microribbons that exhibit overlapping, intertwisting, and parallel formation. This allows them to effectively retain bacterial cells within the spaces and on the surface, thereby significantly improving cell survivability. By contrast, the number of free LGG cells significantly dropped when exposed to AGJ (5.24 log CFU/g) for the same period of time. The presence of prebiotic pullulan provided the highest level of protection to the cells, with a loss of 0.3 log CFU/g. 

These results indicate that granules based on a combined polysaccharide matrix BC/PUL are stable in acid solution, probably due to the interaction between the biopolymer and the prebiotic. A possible underlying mechanism can be that PUL inside the matrix blocks the pores of BC, thereby preventing the diffusion of acidic contents into the granule. Such a protective effect of PUL on probiotic bacteria against gastric conditions has been earlier reported by several authors [45,46,47]. 

Moreover, biofilm is protected by EPS, which enhances microbial survival and safeguards cells from environmental stresses, including acid stress. 

The ingested probiotics reach the target site after passing through the stomach and duodenum. The resistance to the bile acid, which is secreted in the duodenum, is an important characteristic of probiotics. The granules were subjected to 2 h ABJ after AGJ treatment, and the results are shown in Figure 6. 

In spite of the damages and stresses caused by bile salts, LGG is known to tolerate them relatively well, as it was pointed out by Xiao [48]. Indeed, the presence of bile salts in ABJ did not significantly affect the survival of the bacteria in all treatments applied in our study. After 2 h of incubation in ABJ, the viable free cell counts were reduced by 0.6 log CFU/g. In the samples containing BC granules, the number of LGG cells fell by 0.3 log CFU/g, and there was only a slight drop in the viability of cells in BC/PUL by 0.17 log CFU/g after ABJ incubation. 

These findings are in good accordance with data published by Ding et al., who reported that HCl was more harmful to *L. rhamnosus* than ox gall and exposure to acidic conditions resulted in the reduced viability of bacteria [49]. Probiotic strains derived from humans have been found to have greater resistance to bile compared with strains of other origins [50]. Moreover, as mentioned above, the addition of bile to the medium during the immobilization of probiotic cells positively contributed to the biofilm formation and did not cause a reduction in cell number. The concentration of bile in the human intestine varies from 0.2% to 2% [51], which corresponds to the concentration used in the composition of PYG_m_ medium during probiotic immobilization (0.5%).

Overall, for the survivability of LGG cells in BC/PUL granules after gastric and bile juice treatments, there was only a slight difference from the initial number of cells. The data obtained are consistent with the results of Fijałkowski et al. [17], who concluded that the immobilization of lactic acid bacteria on BC provided high-level protection of the bacteria against the negative effect of gastric juice and bile salts.

This approach apparently functions well also when other biopolymers are used as immobilization carriers. Cheow et al. [52] studied the effect of combining alginate with two different types of starch with prebiotic properties, and its potential to enhance the resilience of biofilms. Their research concluded that a modified 0.6% (*w*/*v*) waxy starch coated with chitosan significantly increased the tolerance of an LGG strain biofilm to lyophilization, high temperatures, and cold storage. An earlier report by Doleyres and Lacroix [53] suggests that an immobilized population of bifidobacteria with a high cell density could induce a quorum-sensing response, thereby enabling the adaptation of lactic acid bacteria to changing environmental conditions. The possible reasons for the observed increased resistance are the limited penetration of antimicrobial agents, slower growth rates, activation of the general stress response, induction of a biofilm phenotype, and cell-to-cell communication [29]. The fact that the presence of PUL enhanced resistance implies a synergistic effect of a bacterial biofilm and the supplementary polysaccharide matrix in bacteria protection.

### 3.4. Release of Probiotics into ACJ

The ultimate goal of the LGG immobilization is to ensure their safe delivery into the large intestine. In order to model the large intestinal conditions in vitro, some authors use a system called simulated intestinal juice, which is a solution of electrolytes with a neutral pH [54,55]. Such models, unfortunately, neglect the presence and biochemical activity of huge microbiota (up to 10^13^ CFU/g), consisting of more than 500 species of bacteria alone, not to mention other types of microorganisms [56]. The intestine can be considered as a sophisticated bioreactor constantly working in the human body, in which serious metabolic processes occur associated with the enzymatic activity of microbes living in it, which carry out cavitary digestion [57]. 

Since cellulose granules do not disintegrate in the traditional model of simulated intestinal juice, it was assumed to add fecal enzymatic extract to the solution, which has cellulase activity. 

Traditionally, a commonly used approach involves using a fecal suspension, which includes filtering feces to remove food residues [58]. A similar idea is used in fecal transplantation, a treatment method for GI disorders, particularly Crohn’s disease [59,60]. The fecal suspension contains microorganisms and their metabolic byproducts known as metabiotics, which include cellulolytic enzymes. In this study, microorganisms were removed from the fecal suspension, thereby obtaining an enzymatic fecal extract—fecalase. 

**Figure 7:**
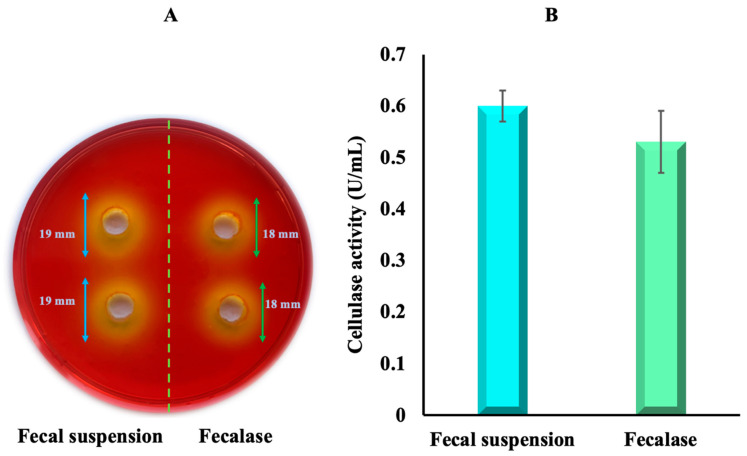
(**A**) CMC hydrolysis zones formed by the fecal suspension and fecalase revealed by Congo red staining, (**B**) evaluation of cellulase activity using the DNS assay.

A semiquantitative analysis was performed to assess the cellulase activity of the fecal suspension and fecalase. Both variants (fecalase and fecal suspension) were introduced into wells that were cut on agar plates containing a substrate—1% CMC. Subsequently, these plates were stained with Congo red solution. Congo red strongly interacts with polysaccharides containing contiguous β-(1-4)-bound-glucopyranosyl units as CMC. The presence of the enzyme was determined by the formation of less colored hydrolysis zones, indicating the presence of cellulase activity (Figure 7A). 

According to the obtained results, the fecal suspension had an enzymatic index E_i_ of 1.8 ± 0.07, while the fecalase had 1.7 ± 0.04. The observed difference in cellulase activity between the samples was, however, not statistically significant (*p* > 0.05). 

In order to better validate the results of the Congo red test, fecalase and fecal suspension were examined with another test for the characterization of cellulase activity, DNS assay. The DNS test is widely used for the detection of cellulase activity and is based on the enzymatic hydrolysis of CMC, leading to the release of reducing sugars. As shown in Figure 7B, using the DNS method, the cellulase activity of both tested groups was also found not significantly different (0.60 ± 0.03 U/mL and 0.53 ± 0.06 U/mL for fecal suspension and fecalase, respectively).

One of our secondary objectives was to compare and choose a sample with the highest cellulase activity. Both methods gave comparable data on fecalase and fecal suspension cellulase activity. If fecal suspension is chosen, then an anaerobic incubator is necessary, since anaerobic bacteria predominate in the normal human fecal flora. Hence, an undoubted advantage of fecalase is that there is no need for an anaerobic incubator, which simplifies the research process. Therefore, fecalase was used in the preparation of ACJ in further experiments.

**Figure 8:**
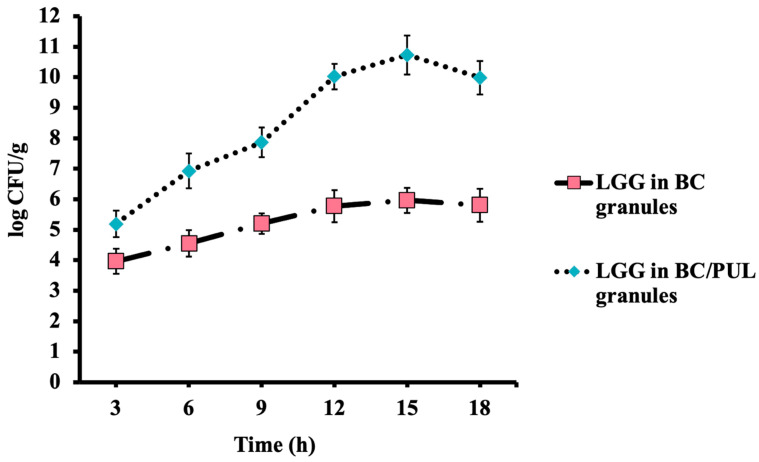
Release of immobilized LGG bacteria into ACJ.

To assess the release of immobilized bacteria following transit through AGJ and ABJ, granules were incubated at 37 °C with gentle shaking in ACJ in the presence of fecalase (Figure 8). 

After 3 h of incubation in ACJ, 3.96 log CFU/g of viable LGG bacteria immobilized in BC granules were released into the bulk medium. This process continued for 15 h, resulting in a final viable cell count of 5.96 log CFU/g, followed by leveling off. 

Compared with the BC group, the LGG release by the BC/PUL granules showed a noticeably higher dynamic. The number of cells that successfully reached the colon not only remained stable but increased by more than 1.5 log (11.4 log CFU/g) compared with the initial value (9.75 log CFU/g). This increase can be attributed to the prebiotic PUL, which served as a carbon source and stimulated the proliferation of probiotic cells, contributing to the formation of a probiotic biofilm. As the prebiotic was digested, the probiotics within the granules were released. Thus, BC/PUL granules facilitated the gradual release of LGG cells, enabling effective colonization in the colon. 

According to our previous study [61], the encapsulation of LGG in BC/PUL beads allowed bacteria to be safely delivered into ACJ up to 10.2 log CFU/g. Based on the data obtained, the biofilm-forming incubation method of immobilization used in this work is more efficient, as 11.4 log CFU/g of LGG was detected in ACJ, which is 10 times higher. This technique offers a more advantageous and cost-effective approach, presenting a simpler and more economical technological method of probiotic immobilization.

Similar data were obtained in the next series of experiments, where animals were used as an experimental model.

These experiments showed that BC/PUL granules were successfully degraded in the model colon, which allows for considering them as a suitable carrier to deliver probiotics. The release of LGG occurred due to the combination of cellulase activity and the peristaltic movement in the lower GI tract, resulting in the swelling of the cellulose granules and their degradation. Thus, a polysaccharide matrix (BC/PUL) with probiotic biofilms (LGG) was further studied in the next (in vivo) part of the work.

### 3.5. In Vivo Determination of Viable LGG Counts in Fecal Samples of Laboratory Rats

The real activity and effectiveness of exogenous probiotic strains can be assessed through the ability to survive in vivo. This is due to the fact that the clinical effectiveness of probiotics is linked to the colonization of the intestinal mucosa and the replacement of functions, which ensures the establishment of normal indigenous flora. Thus, it creates a microecological environment that promotes the correction of the native microflora. 

During the colonization of the intestine, LGG displays higher adhesion activity compared with many related strains, which can be attributed to the presence of specific pili [62]. When LGG attaches to the intestinal epithelium, it inhibits the adhesion of other microbes, a phenomenon known as competitive colonization. This ability is probably a key factor underlying LGG’s effectiveness in preventing intestinal infections [63]. 

The ability of probiotics to survive in the human GI tract should result in the presence of live cells being shed in fecal samples. However, survival depends on both the specific probiotic strain and the matrix involved. To confirm the findings of the in vitro observations that indicated sufficient protection of immobilized bacteria from acid and bile exposure and their release in a colon model, PMPB granules were administrated to rats. Rats ingested a PMPB supplement containing at least one billion CFU per dose. 

**Figure 9:**
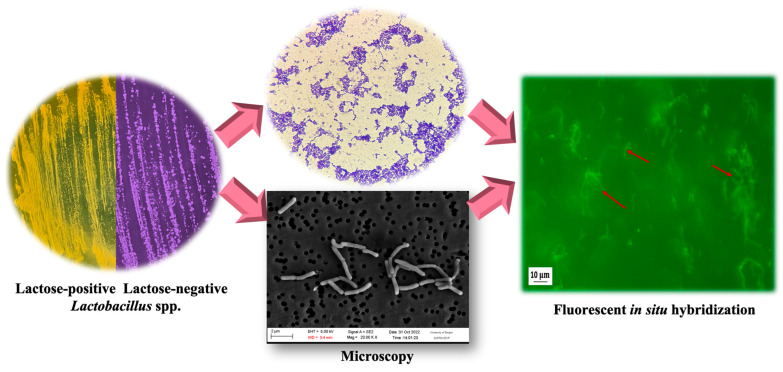
LGG identification after GI transit in rats: colony culturing on MRS medium with indicator; microscopy (conventional light microscopy, SEM); FISH using a target-specific probe for 16S rRNA sequence.

Fecal samples were collected at different time intervals during and after probiotic intake and cultured on a nutrient medium for analysis. They contained a large species diversity of lactobacilli. To detect LGG specifically, we used a scheme consisting of (a) seeding of presumptive colonies on MRS medium with indicator, (b) microscopy, and (c) the FISH technique (Figure 9).

LGG differs from most other lactic acid bacteria by its inability to ferment lactose [64], which was used for their identification by selecting typical LGG-like colonies from each fecal sample and cultivating them on lactose MRS agar with indicator dye. Lactose-negative bacteria did not change the medium color from purple to yellow, suggesting that they may belong to LGG. Lactose-positive bacteria changed the color of the medium due to lactic acid production by lactose fermentation. It was also observed that the selected colonies were Gram-positive, rod-shaped bacteria forming long chains typical of the strain GG. 

FISH is on the rise for the detection of probiotic bacteria that are intentionally administered to the GI tract [65]. In our study, the fluorescently labeled specific probe Lcas467-Cy3 was hybridized with the selected lactose-negative typical LGG-like bacteria. The hybridized bacteria showed fluorescence intensity in the microscopic image. Based on the results of FISH, all of the analyzed colonies from each fecal sample belonged to LGG. As can be seen from Figure 9, this technique allowed for studying the bacterial cell morphology and showed typical long chains of GG. 

When consuming both free and immobilized cells, they were determined in the feces on the second day, reaching a maximum on the third day (Table 2). At the same time, the titer of cells varies significantly, depending on the form in which they pass through the GI tract of animals.

Overall, we found the highest concentration of viable immobilized LGG in fecal samples obtained between 3 and 7 days after the beginning of intake (7.38–7.63 log CFU/g). After the introduction of free cells, their maximum number in feces ranged between 3.09 and 3.51 log CFU/g of feces. LGG colonies were not detected in the control rats due to the absence of this strain in these animals’ intestinal ecosystems.

The results indicate a better preservation of cell viability in the immobilized state and their unhindered passage through the upper parts of the digestive tract. After stopping the intake of nonimmobilized probiotics, the concentration of exogenous lactobacilli in feces decreased until complete disappearance after 10 days. The data obtained show that with this method of delivery, only short-term transient colonization of the intestine by exogenous lactobacilli occurs. At the same time, with the introduction of PMPB granules, 5.12 log CFU/g was detected even a week after the last intake.

## 4. Conclusions

In summary, the PMPB immobilization proved to be effective, leading to the successful recovery of viable LGG from all the animals enrolled in the study. It demonstrates that the polysaccharide matrix, containing at least one billion CFU, is a suitable carrier for successfully delivering probiotic cells to the colon. The interpenetrating pores in the cellulose matrix create an open cell structure that leads to the premature release of probiotic cells in the stomach during passage through the upper GI tract. The addition of PUL allowed for overcoming this limitation, presumably due to sealing the pores, which led to better survival and later a higher release of the LGG. 

The collected experimental data obtained allow us to argue that BC acted as a spacious “shelter” capable of accommodating a significant number of LGG cells, and PUL acts as a prebiotic, stimulating the growth of probiotic cells and the formation of a biofilm by them. A bacterial biofilm presumably serves as a protective “barrier” that protects these shelters. This barrier remained closed when faced with adverse conditions, such as gastric acid and bile salts. However, it opened up upon reaching the target site (the colon), enabling the probiotic cells to effectively colonize the intestine.

The proposed system, consisting of a neutral carrier, prebiotic, and probiotic, represents an interesting approach aimed at enhancing the effectiveness of existing probiotics and holds potential for future probiotic applications.

## Figures and Tables

**Table 1:** Effect of EPS-induced components on biofilm formation.

Concentration (g/L)	OD 570	
	Inulin	Bile
0.5	2.8 ± 0.3	4.2 ± 0.8
1	3.7 ± 0.4	4.4 ± 0.3
2	3.5 ± 0.1	3.5 ± 0.5

OD—optical density; the data are the average OD values ± the standard error of the mean.

**Table 2:** Viable counts of LGG (log CFU/g) in the fecal samples of rats.

**Day**	**Free LGG**	**PMPB**
	During Consumption	
1	0	0
2	2.05 ± 0.13	5.32 ± 0.23
3	3.09 ± 0.18	7.63 ± 0.11
4	3.34 ± 0.31	7.39 ± 0.21
5	3.51 ± 0.54	7.44 ± 0.25
6	3.49 ± 0.19	7.41 ± 0.13
7	3. 31 ± 0.23	7.38 ± 0.32
	After consumption	
8	2. 53 ± 0.13	7.29 ± 0.35
9	1.76 ± 0.22	6.54 ± 0.54
10	1.04 ± 0.09	6.04 ± 0.42
11	0	5.95 ± 0.12
12	0	5.53 ± 0.65
13	0	5. 32 ± 0.45
14	0	5.12 ± 0.58

PMPB—polysaccharide matrix with probiotic biofilms; the data are the average log CFU/g of feces ± the standard error of the mean.

## Data Availability

Data are contained within the article.
